# New quantitative classification of the anatomical relationship between impacted third molars and the inferior alveolar nerve

**DOI:** 10.1186/s12880-015-0101-0

**Published:** 2015-12-07

**Authors:** Wei-Quan Wang, Michael Y. C. Chen, Heng-Li Huang, Lih-Jyh Fuh, Ming-Tzu Tsai, Jui-Ting Hsu

**Affiliations:** School of Dentistry, College of Medicine, China Medical University, 91 Hsueh-Shih Road, Taichung, 40402 Taiwan; Department of Dentistry, China Medical University and Hospital, Taichung, 404 Taiwan; Department of Bioinformatics and Medical Engineering, Asia University, Taichung, 413 Taiwan; Department of Biomedical Engineering, Hungkuang University, Taichung, 433 Taiwan

**Keywords:** Impacted third molar, Inferior alveolar nerve, Computed tomography, Cartesian coordinate system, Cylindrical coordinate system

## Abstract

**Background:**

Before extracting impacted lower third molars, dentists must first identify the spatial relationship between the inferior alveolar nerve (IAN) and an impacted lower third molar to prevent nerve injury from the extraction. Nevertheless, the current method for describing the spatial relationship between the IAN and an impacted lower third molar is deficient. Therefore, the objectives of this study were to: (1) evaluate the relative position between impacted lower third molars and the IAN; and (2) investigate the relative position between impacted lower third molars and the IAN by using a cylindrical coordinate system.

**Methods:**

From the radiology department’s database, we selected computed tomography images of 137 lower third molars (from 75 patients) requiring removal and applied a Cartesian coordinate system by using Mimics, a medical imaging software application, to measure the distribution between impacted mandibular third molars and the IAN. In addition, the orientation of the lower third molar to the IAN was also measured, but by using a cylindrical coordinate system with the IAN as the origin.

**Results:**

According to the Cartesian coordinate system, most of the IAN runs through the inferior side of the third molar (78.6 %), followed by the lingual side (11.8 %), and the buccal side (8.9 %); only 0.7 % is positioned between the roots. Unlike the Cartesian coordinate system, the cylindrical coordinate system clearly identified the relative position, *r* and *θ*, between the IAN and lower third molar.

**Conclusions:**

Using the cylindrical coordinate system to present the relationship between the IAN and lower third molar as (*r*, *θ*) might provide clinical practitioners with a more explicit and objective description of the relative position of both sites. However, comprehensive research and cautious application of this system remain necessary.

## Background

In the clinical environment of oral medicine, extraction of the lower third molar is a common surgical procedure. However, various postsurgery complications can occur [1–5]. Among such complications, injury to the inferior alveolar nerve (IAN) is the most severe. Because the IAN occasionally contacts or is near the third molar/root, or molars with a “crooked root,” the IAN can be easily damaged during this procedure. Temporary and permanent IAN injury comprises approximately 5.0–7.0 % and 0.5–1.0 % of such incidents, respectively [2, 6–12]. Therefore, information regarding the spatial relationship between the third molars and IAN is critical for preoperative procedures.

Clinically, taking panoramic film is essential for evaluation before extraction. However, because panoramic film is a two-dimensional imaging tool, the image can be distorted or overlapped [13]. Nakagawa et al. [14] stated that this may lead clinicians to misinterpret the results or make incorrect judgments. Therefore, to minimize postoperative complications and improve judgments, computed tomography (CT) is introduced to evaluate the spatial relationship between the third molar and IAN canal.

Based on the Cartesian coordinate system concept, Maegawa et al. [15] reported that the probability of the IAN canals being positioned at the buccal, lingual, and inferior sides of the lower third molar and between the roots was 51, 26, 19, and 4 %, respectively. Ghaeminia et al. [16] employed a similar method on Westerners and reported observations on the buccal side (17 %), lingual side (49 %), inferior side (19 %), and between the roots (15 %). In addition, various researchers have reported inconsistent data including the trends of position probability. This is primarily because of measurement position discrepancies among various races; it may also have resulted from differences in the tooth morphology of the mandible structure and lower third molar root, which can cause approximate and imprecise classification of the teeth (such as between the inferior and buccal and between the inferior and lingual), leading to judgment errors and varying results.

Accordingly, this study was conducted to analyze the spatial relationship between impacted third molars and the IAN, the results of which were compared with those of previous studies. Specifically, this study was aimed at (1) establishing the distribution between impacted third molars and the IAN, and (2) investigating the relative position between the lower third molars and IAN by using a cylindrical coordinate system.

## Methods

### Patient selection

This retrospective study was based on a database of head CT scans for patients presenting to the Radiology Department of the China Medical University Hospital for an evaluation of impacted mandibular third molars and the IAN between July 2009 and January 2011. In this retrospective study, only the mandibles of patients with impacted third molars were selected for measurement. CT scans were performed (LightSpeed, General Electric, Milwaukee, WI) with the following technical parameters: 1.25-mm increments, 240 mm field of view, and 512 × 512 pixels. Before the position of the IAN was measured, the patient’s head was first rotated with the Down’s mandibular plane parallel to the horizontal plane in Mimics, then further rotated with the mandible centered on the midsagittal plane of the image perpendicular to the Down’s mandibular plane. Subsequently, buccolingual images were created using the “online reslice” function of Mimics to obtain continual slices of the mandibular bone. There was no need to obtain consent because this is a retrospective study. In addition, all the patients were adults. The research protocol was approved by the institutional research board of China Medical University and Medical Center.

### Measurements of the relationship between the IAN and third molar

#### Cartesian coordinate system

**Fig. 1:**
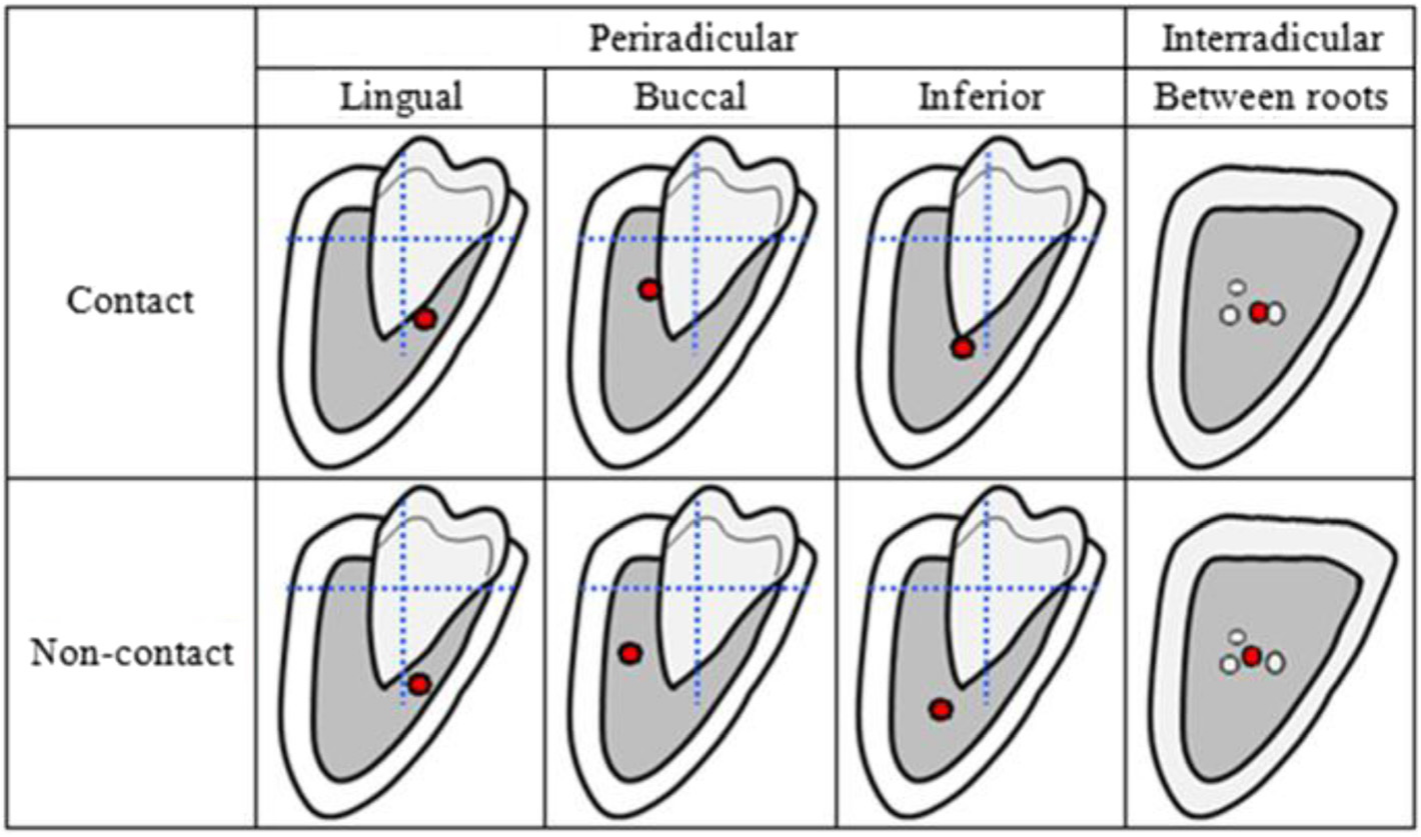
Classification of the position and relationship of the IAC with the right mandibular third molars using the Cartesian coordinate system

From continuous buccolingual slices, the layer where the IAN and lower third molar were the closest was selected as the reference image to determine their relative position. First the position of the IAN and lower third molar were categorized as either contacting or noncontacting, and then the structural center of the lower third molar was located to serve as the origin in the Cartesian coordinate system. Subsequently, we determined whether the IAN was distributed on the lingual side, buccal side, or inferior side of the lower third molar or between the roots (Fig. 1).

#### Cylindrical coordinate system

**Fig. 2:**
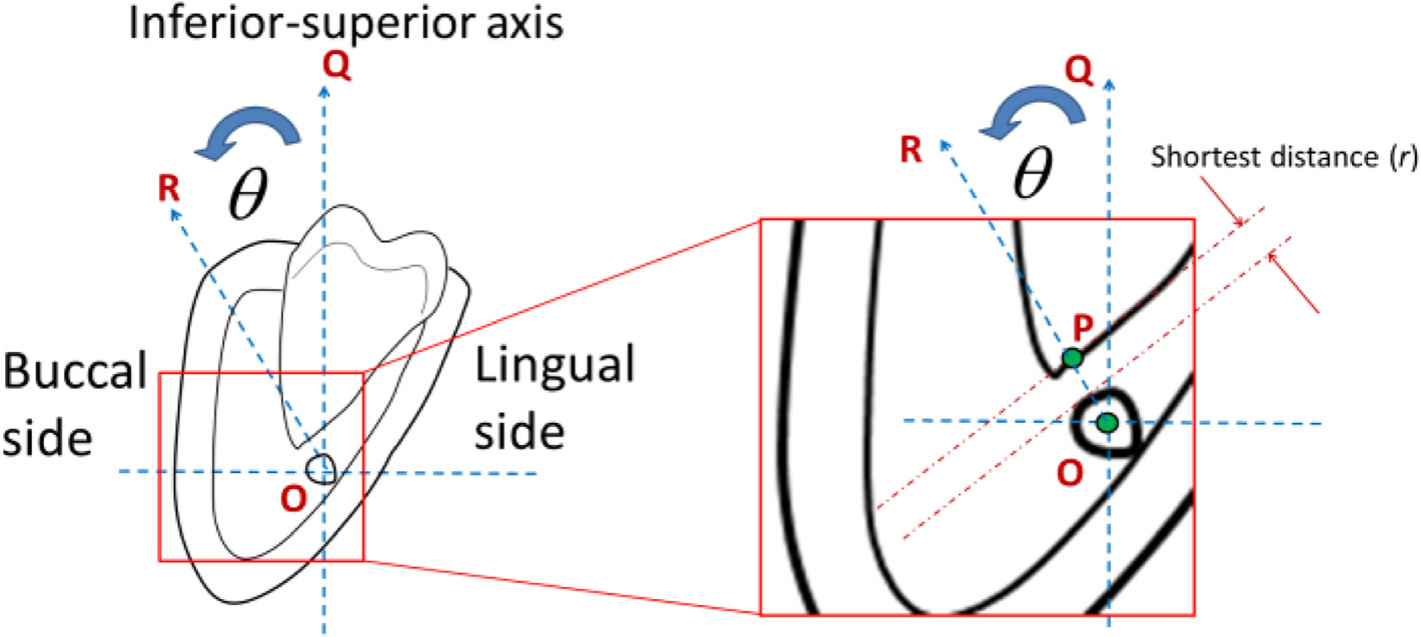
Classification of the position and relationship of the IAN canal with the right mandibular third molars using the cylindrical coordinate system. This approach can be defined as follows: (1) Use the IAN bundle as the original reference point (point O); (2) find the point on the third molar (point P) closest to the IAN bundle, and draw a line (line OR) from the original reference point (point O) to point P; and (3) determine the angle between the inferior-superior axis (line OQ) and line OR

From continuous buccolingual slices, the layer where the IAN and lower third molar were the closest was selected as the reference image to evaluate their relative position. First, the position of the IAN and lower third molar were categorized as contacting or noncontacting, and then the IAN (the structural center of the IAN canal) was located to serve as the origin in the cylindrical coordinate system. A line was drawn from this point to the nearest point on the lower third molar root, and the angle between this line and the inferior–superior axis was measured from 0° to 360° and then divided by 30°. The shortest distance between the lower third molar and the mandible canal was also measured (Fig. 2). The relationship between the IAN and lower third molar can be expressed as (*r*, *θ*) in the cylindrical coordinate system, where *r* is the shortest distance between the mandible and lower third molar, and *θ* is the orientation of the lower third molar relative to the IAN.

### Statistical analysis

Descriptive statistics were computed to classify the probability distribution by using either the Cartesian coordinate system or cylindrical coordinate system.

## Results

Based on the selection criteria, mandibular bone CT images of 75 patients (age_mean_ 37.0 + 18.9 years; age_range_ 18–77 years) were used in this study. From the 75 mandibular bones, 137 lower third molars (100 noncontact cases; 37 contact cases) were used for the measurement. For the Cartesian coordinate system (Table 1), the IAN distribution relative to the third molar was 78.8 % (108/137) in the inferior position, 11.7 % (16/137) in the lingual site, 8.8 % (12/137) in the buccal site, and 0.7 % (1/137) between the roots (Table 1).

**Table 1:** Relationship between the IAN and third molar using the Cartesian coordinate system

Anatomical relationship	Contact	Non-contact	Total	Percentage (%)
Buccal	3	9	12	8.8
Lingual	8	8	16	11.7
Between roots	1	0	1	0.7
Inferior	25	83	108	78.8
Total	37	100	137	100

Regarding the relationship between the IAN and third molar in an angular distribution, no case between 60° and 270° was observed using the cylindrical coordinate system (Table 2). The highest probability region with 43.8 % was between 330° and 360°, followed by 32.1 % between 0° and 30°, and 21.2 % between 300° and 330°. The lowest probability with 0.8 % was between 270° and 300°. In addition, for the noncontact cases, Table 3 lists the shortest distances between the mandible canal and third molar. Most of them occurred above the 3-mm group, 11 % were in the 1–2-mm group, 11 % were at 1–2 mm, and only 3 % were in the 0–1-mm group.

**Table 2:** Orientation of the lower third molar in relation to the IAN using the cylindrical coordinate system

Anatomical relationship	Contact	Non-contact	Total	Percentage (%)
0 ~ 30°	23	21	44	32.1
30° ~ 60°	1	2	3	2.1
60° ~270°	0	0	0	0
270° ~ 300°	1	0	1	0.8
300° ~ 330°	6	23	29	21.2
330° ~ 360°	6	54	60	43.8
Total	37	100	137	100

**Table 3:** The shortest distance between the mandible canal and the lower third molar for non-contact cases

The nearest distance	Sample number	Percentage (%)
0 ~ 1 mm	3	3
1 ~ 2 mm	11	11
2 ~ 3 mm	11	11
>3 mm	75	75
Total	100	100

## Discussion

To reduce the risk of IAN injury when extracting the mandibular third molars, the appropriate tools must be used. Therefore, using CT images to accurately identify the relationship between the molars and IAN at the buccolingual section is crucial. In previous studies, the reported probability of various IAN positions relative to the lower third molars has been inconsistent. This is possibly due to the human discrimination affects derived from the subjective judgments of different dentists. The morphologies of the alveolar bone and lower third molar are highly divergent, which easily leads to approximate and imprecise distinguishing and classification (such as between the inferior and buccal sides and between the inferior and lingual sides). This eventually results in judgment errors and inconsistent results. Compared with the traditional Cartesian coordinate system, the anatomical structure can be categorized more objectively and accurately by using the cylindrical coordinate system. This study developed a method that involves using a cylindrical coordinate system to assess the relationship between the IAN and lower third molar for Asian populations.

Previous studies have compared the ability of CT and panoramic filming for evaluating the relative position of the IAN and third molar [15, 17]. These studies have indicated that CT is an efficient tool for determining the relative position between the IAN and lower third molar. Although CT scans were employed in determining the relative position, we varied from other studies [15, 17, 18] in that the Down’s mandibular plane was employed because resectioning methods cause the buccolingual section slices to become perpendicular to the mandibular canal.

In recent years, dental cone-beam CT (CBCT) has been prevalently employed in dental surgery [19–21] and basic research [22–25]. In addition to the presurgical orthodontic, orthognathic, and endodontic treatment evaluations, CBCT images also frequently serve as a reference when extracting impacted lower third molars [16, 26]. Therefore, although a CT image database was employed in the present study, the proposed cylindrical coordinate system proposed can also be applied to CBCT images.

The spatial relationships between the IAN and lower third molar identified in this study were compared with those reported in literature (Table 4). When the Cartesian coordinate system was used, the IAN distribution relative to the third molars was found to be consistent with that reported by Tantanapornkul et al. [26] (Table 4). The highest distribution was on the inferior side, and the probability distribution on the lingual side was slightly higher than that on the buccal side; the lowest distribution was between the roots. Similarly, Monoco et al. [9] also indicated that the probability that the IAN would be located on the inferior side was the highest (Table 4). However, Ueda et al. [27] and Maegawa et al. [15] reported that the probability of the IAN distribution on the buccal side was higher than that on the lingual side (Table 4). Ghaeminia et al. [16], de Melo Albert et al. [28], and Ohman et al. [17] have reported that the probability of the distribution on the lingual side was the highest (Table 4). Variations in the results of previous studies and those of the present study may be due to the differing patient ethnicities of the patients and the various orientations of the mandibular bone in the buccolingual slices.

**Table 4:** The buccolingual distribution of the IAN in relation to the lower third molar, as reported in previous studies

	This study	Ueda et al. [27]	Ghaeminia et al. [16]	Tantanapornk-ul et al. [26]	De Melo Albert et al. [28]	Ohman et al. [17]	Monoco et al. [9]	Maegawa et al. [15]
Buccal	8.9 %	45.5 %	17 %	25 %	45 %	31 %	25 %	51 %
Lingual	11.8 %	32.4 %	49 %	26 %	48 %	33 %	19 %	26 %
Between roots	0.7 %	0.7 %	15 %	4 %		10 %	5 %	4 %
Inferior	78.6 %	21.4 %	19 %	45 %	7 %	26 %	51 %	19 %
Number	136	145	53	142	31	90	73	47

Regarding the experimental results of previous studies, Maegawa et al. [15], Tantanapornkul et al. [26], and Ueda et al. [27] have investigated conditions in Japan, which is near Taiwan. However, based on the Cartesian coordinate system, the spatial distributions in the experimental results of these studies are inconsistent. Maegawa et al. [15] and Ueda et al. [27] have reported that the highest distribution was on the buccal side, followed by the lingual side, inferior side, and then between the roots. The experimental results of the current study were similar to those reported by Tantanapornkul et al. [26], which indicated that the highest distribution was on the inferior side, followed the lingual side, buccal side, and then between the roots. This difference may have resulted from the ambiguous classification derived from the Cartesian coordinate system, particularly in the buccoinferior and linguoinferior regions.

Although the cylindrical coordinate system is used in orthodontics [29], it is not widely employed for oral surgery. During extraction of the lower third molar, protecting the IAN is critical. This raises questions as to why observations are made from the perspective of the molar to be extracted and why the position of the nerve is categorized relative to the molar. Instead, we propose setting the IAN requiring protection as the origin and then observing the relative position of the nearby molar. Therefore, in the present study, we used a cylindrical coordinate system to categorize the relationship between the IAN and lower third molar. The advantage of using the cylindrical coordinate system is that it simplifies classifying the relationship between the third molars and IAN. The two parameters identified by this system—namely, the relative angle and shortest distance between the mandible canal and third molars—provide quantitative data and a detailed understanding for further investigation. The angle data not only reveal the distribution between the IAN and lower third molar, but may also prevent misunderstanding among clinical practitioners and researchers. Furthermore, the shortest distance between the lower third molar and mandible canal provides additional information regarding the distance between the mandible canal and third molar root, which is not available when using the Cartesian coordinate system. The shorter the distance is between these two structures, the greater the possibility for IAN injury during extraction of the lower third molar. Therefore, determining this distance is crucial. As shown in Table 3, an increment of 1 mm was adopted as the scale. Our observations indicated that for 75 % of all cases (75 cases), the distance between the IAN and lower third molar exceeded 3 mm, and for 3 % of cases (3 cases) the distance was shorter than 1 mm. In two of these three cases, removing the impacted third molars presented greater difficulty (the IAN was located on the lingual side relative to the lower third molar); thus, removal of impacted lower third molars necessitates additional caution.

To prevent situations where whether the IAN is located in the buccal or inferior region (or in the lingual or inferior region) cannot be determined, the position (*r*, *θ*) of the IAN relative to the lower third molar can be determined using the cylindrical coordinate system. Although Miller et al. [30] proposed inferior–lingual and inferior–buccal classifications regarding the relationship between the IAN and lower third molars, communication (particularly verbal) between clinical practitioners remain vague; by comparison, the cylindrical coordinate system (*r*, *θ*) provides superior clarity.

This study was subject to several limitations. First, all human samples were of Asian ethnicity, and whether the differences would occur in other ethnic groups was not explored. Second, the effects of gender and age were not investigated because of the small sample. Third, given the current status of the cylindrical coordinate system, the segmentation of impacted lower third molars was based on a manual approach. In future, an automated approach should be developed to enhance the clinical application of this system. Finally, unlike the prevalent use of the Cartesian coordinate system, the cylindrical coordinate system remains a new method for analyzing the relationship between the IAN and lower third molar. Comprehensive investigations are necessary to further verify the academic results and clinical applicability of this approach.

## Conclusion

This study proposes a cylindrical coordinate system as a new analysis system for clarifying the anatomical relationship between the lower third molars and IAN. The Cartesian coordinate system revealed that the highest distribution is on the inferior side (78.6 %), followed by the lingual side (11.8 %), buccal side (8.9 %), and then between the roots (0.7 %). Based on the results of the study, using the cylindrical coordinate system to present the relationship between the IAN and lower third molar as (*r*, *θ*) might provide clinical practitioners with a more explicit and objective description of the relative position of both sites. However, comprehensive research on and cautious application of this system in the future remain necessary.

## Abbreviations

IAN: Inferior alveolar nerve
CT: Computed tomography
